# Impact of annual wellness visits on preventing falls and fractures for Alzheimer’s disease and related dementias older adults

**DOI:** 10.1093/ageing/afag065

**Published:** 2026-03-30

**Authors:** Sheheryar Ali, Yong-Fang Kuo, Yong Shan, Huey-Ming Tzeng, Mukaila A Raji

**Affiliations:** The University of Texas Medical Branch John Sealy School of Medicine, Galveston, Texas, USA; The University of Texas Medical Branch at Galveston School of Public and Population Health, Galveston, Texas, USA; The University of Texas Medical Branch at Galveston, Department of Internal Medicine-Geriatric Medicine Division, Galveston, Texas, USA; The University of Texas Medical Branch at Galveston, Sealy Center on Aging, Galveston, Texas, USA; The University of Texas Medical Branch at Galveston, Office of Biostatistics, Galveston, Texas, USA; The University of Texas Medical Branch at Galveston School of Public and Population Health, Department of Biostatistics and Data Science, Galveston, Texas, USA; The University of Texas Medical Branch at Galveston, Office of Biostatistics, Galveston, Texas, USA; The University of Texas Medical Branch at Galveston School of Public and Population Health, Department of Biostatistics and Data Science, Galveston, Texas, USA; The University of Texas Medical Branch at Galveston, Sealy Center on Aging, Galveston, Texas, USA; The University of Texas Medical Branch at Galveston School of Nursing, Galveston, Texas, USA; The University of Texas Medical Branch at Galveston, Sealy Center on Aging, Galveston, Texas, USA; The University of Texas Medical Branch John Sealy School of Medicine, Department of Internal Medicine-Geriatric Medicine Division, Galveston, Texas, USA

**Keywords:** Alzheimer’s disease and related dementias, annual wellness visit, fall prevention, fracture prevention, health services, older people

## Abstract

**Background:**

Falls and fractures are leading causes of disability and premature death among older adults with Alzheimer’s Disease and Related Dementias (ADRD). Annual Wellness Visits (AWVs), which Medicare reimburses, can screen and manage fall/fracture-related risk factors. This study evaluates the association between AWV and falls/fractures prevention among Medicare beneficiaries with ADRD.

**Methods:**

We analysed a cohort of Medicare beneficiaries in 2017 aged ≥68 years with ADRD and continuous enrollment from 2014 to 2016 (*n* = 1 610 637). AWV recipients were stratified by the number of visits before 2017 (0, 1, 2 or ≥ 3). Kaplan–Meier methods estimated rates of falls and fractures. Patients were censored at end of study (12/31/2021), if they lost Medicare coverage, or switched to HMO. We used inverse probability treatment weighting (IPTW) with propensity score in Cox proportional hazards models to assess the effect of AWVs.

**Results:**

AWVs were associated with reduced risks for falls (HR: 0.976 for 2 AWVs; 0.936 for ≥3 AWVs) and fractures (HR: 0.978 for 1 AWV; 0.960 for 2 AWVs; 0.927 for ≥3 AWVs), with greater reductions observed with more AVWs. Time-dependent models revealed AWV in follow-up period had stronger effects on fall and fracture risk (HR: 0.921 and 0.929, respectively). In subgroup analyses, AWV risk reduction was weaker for falls in Black and rural residents with no significant fracture risk reduction.

**Conclusions:**

Our studies indicate more AWVs are associated with greater risk reduction of falls/fractures in older adults with ADRD. This study emphasizes expanding awareness of AWVs to prevent falls/fractures in this population.

## Key points

- Annual Wellness Visits (AWVs) are linked to reduced falls and fractures in older adults with Alzheimer’s Disease and Related Dementia (ADRD).
- AWVs can be used a way to improve fall and fracture prevention for older adults with Alzheimer’s and Dementia.
- More frequent AWVs are associated with greater reductions in fall and fracture risk for older adults with ADRD.
- AWVs are a vital preventative health tool for older adults with ADRD.

## Introduction

Falls and fractures are common and potentially modifiable contributors to excess disability, recurrent emergency department visits, hospitalizations, nursing home placement and premature deaths in older adults especially those with Alzheimer’s Disease and Related Dementia (ADRD) [1–4]. Falls and fractures also contribute to diminished quality of life, caregiver stress and increase in healthcare costs [5]. With the ageing population comes a rapid increase in populations of older adults living with ADRD, underscoring the urgent need to develop strategies to prevent falls and fractures in this group. Past intervention trials (e.g. physical exercise, medication reviews and environment modifications) aimed at reducing fall and fracture risk in ADRD populations have yielded mixed results [6, 7]. Clinical guidelines, such as the World Falls Guidelines and the CDC STEADI fall-prevention recommendations, emphasise structured fall-risk screening, medication review, gait and balance assessment and home-safety interventions [8, 9].

In 2011, the Centers for Medicare and Medicaid Services (CMS) started reimbursing Annual Wellness Visits (AWVs), a preventive healthcare service that conducts fall risk screenings and other preventive measures, such as detecting cognitive impairment, reviewing functional ability and safety [10]. While AWVs are associated with a reduced risk of falls and fractures in the general population of older adults it is unclear if the association extends to the ADRD population. There is thus a gap in knowledge on the relationship of AWV participation and risk of fall and fracture in older adults with ADRD [5, 10, 11].

Our study addresses this gap by examining the association between AWV participation and the risk of falls and fractures among Medicare beneficiaries with ADRD. By leveraging a large national cohort of Medicare beneficiaries, our research aims to clarify the role of AWVs in preventing falls and fractures. We hypothesise that AWVs will be associated with reduced risk of falls and fractures, with effect size varying by severity of ADRD, social determinants of health and frequency of AWVs. Specifically, we predict that a higher frequency of AWVs will lead to a greater reduction in the risk of fall and fracture in the ADRD population.

## Methods

### Data source

We used Medicare data from 100% beneficiaries with diagnosis of ADRD in 2017 through access to the CMS Virtual Research Data Centre. We used the Master Beneficiary Summary File to determine beneficiary demographic characteristics. Outpatient AWVs were determined using the Outpatient Standard Analytical files and Carrier files. Fall and fracture were determined using the Medicare Provider Analysis and Review files, Outpatient Standard Analytical files and Carrier files. Fall and fracture claims are documented medical encounters recorded by clinicians for billing purposes and are not self-reported by patients The institutional review board of the University of Texas Medical Branch (IRB #23–0171) approved this study.

### Cohort selection

ADRD diagnosis was based on a validated claims-based method by using CMS 27 Chronic Conditions Warehouse (CCW27) Algorithms [12]. We divided the ADRD patients into incidence ADRD and prevalence ADRD. The incidence ADRD was first diagnosed in 2017 and prevalence ADRD is first diagnosed before 2017. The diagnosis date of ADRD in 2017 is the index date for incidence ADRD, and we set 1/1/2017 as the index date for prevalence ADRD. We kept the cohort as patient ≥68 years old and restricted the study population to beneficiaries with complete Medicare A and B and no Medicare Advantage enrolment from three years before the index date to the death date or 12/31/2021, which one comes first. Our final cohort included 1,610,637 patients.

### Medicare AWVs

Medicare allows one AWV every 12 months for beneficiaries to have Medicare Part B medical insurance for >12 months and with no initial preventive physical examination or AWV providing a personalised prevention plan within the preceding 12 months. Beneficiaries have no co-payment if a healthcare provider accepts the assignment. Healthcare providers can bill G0438 (for an initial AWV) or G0439 (a subsequent AWV) for the serviced delivered to a beneficiary once each year. We calculated the number of previous AWVs the three years before the index date (0,1,2,3+).

### Outcomes

We have two outcomes fracture and fall (Supplementary Table  1). We identified fracture from primary ICD 10 diagnosis code and fall from any ICD-10 diagnosis codes from inpatient, outpatient or carrier claims after the index AWV through 12/31/2021.

### Covariates

The beneficiary characteristics obtained for the analysis included demographics (age, gender identity, race/ethnicity and residential area), original Medicare entitlement, Medicaid, fall or fractures that occurred in the 12 months preceding the index AWV, osteoporosis and tobacco use that occurred in the 2 years before the index date. We categorized moderate/severe ADRD based on frailty index score at cut-off of 0.28 [13]. We extracted two county-level covariates (with quartile 1 the lowest and 4 the highest): physical inactivity and social opportunities; and one Zip code-level covariate: high school graduation rate. We included four medical service utilisation characteristics in the 12 months before cognitive impairment diagnosis: (i) number of hospitalizations (0, 1, 2, 3, ≥4); (ii) number of neurologist visits; (iii) number of psychiatrist visits; and (iv) having a primary care provider (PCP).

### Statistical analysis

Kaplan–Meier curves were generated for fall and fractures by the number of AWVs. Beneficiaries were censored at loss of Medicare coverage, death, or end of study (12/31/2021). We performed proportion hazards models including the number of AWVs (0, 1, 2, 3+) for fracture and fall.

To control selection bias with previous AWVs (0,1,2,3+), we applied Inverse Probability of Treatment Weighting (IPTW) on it. All patient covariates in Table 1 were used in a multinomial logit model to predict the probability of AWV use. We then calculated the weights as the inverse of the propensity score. Standardised differences were calculated to assess the balance of covariates between the groups before and after IPTW. A standardised difference of less than 0.10 between groups indicated a balance in covariates. All covariates are balanced well, except for ‘having a PCP’. We performed Cox proportional hazard model with the IPTW weight for fracture and fall, and hazard ratio (95% CI) were represented. We used Schoenfield residuals to evaluate the Proportional Hazard (PH) assumption.

**Table 1:** Characteristics of 1 610 637 Medicare beneficiaries before and after IPTW.

**Characteristic**	**Overall (N)**	**0 AWV**		**1 AWV**		**2 AWV**		**>3 AWV**		**Std. Dif.**	
		**(n)**	**(%)**	**(n)**	**(%)**	**(n)**	**(%)**	**(n)**	**(%)**	**Pre-IPTW**	**Post-IPTW**
Beneficiaries	1 610 637	1 065 772	(66.2%)	287 646	(17.9%)	170 047	(10.6%)	87 172	(5.4%)		
ADRD Prevalence											
No	555 722	355 313	(33.3%)	99 223	(34.5%)	64 437	(37.9%)	36 749	(42.2%)	0.1827	0.0472
Yes	1 054 915	710 459	(66.7%)	188 423	(65.5%)	105 610	(62.1%)	50 423	(57.8%)	0.1827	0.0472
Group											
Mild	1 058 779	681 397	(63.9%)	191 395	(66.5%)	119 469	(70.3%)	66 518	(76.3%)	0.2728	0.072
Severe	551 858	384 375	(36.1%)	96 251	(33.5%)	50 578	(29.7%)	20 654	(23.7%)	0.2728	0.072
Sex											
Female	985 068	649 604	(61.0%)	180 134	(62.6%)	104 342	(61.4%)	50 988	(58.5%)	0.0846	0.0095
Male	625 569	416 168	(39.0%)	107 512	(37.4%)	65 705	(38.6%)	36 184	(41.5%)	0.0846	0.0095
Age at diagnosis											
68–74	267 321	174 479	(16.4%)	48 656	(16.9%)	28 939	(17.0%)	15 247	(17.5%)	0.0299	0.0076
75–79	293 117	187 978	(17.6%)	53 876	(18.7%)	33 286	(19.6%)	17 977	(20.6%)	0.0759	0.0111
80–84	350 934	228 131	(21.4%)	63 664	(22.1%)	38 692	(22.8%)	20 447	(23.5%)	0.0492	0.0099
>85	699 265	475 184	(44.6%)	121 450	(42.2%)	69 130	(40.7%)	33 501	(38.4%)	0.1252	0.0216
Race											
Non-Hispanic white	1 387 908	911 025	(85.5%)	250 347	(87.0%)	149 235	(87.8%)	77 301	(88.7%)	0.0954	0.0217
Hispanic	60 952	42 111	(4.0%)	10 564	(3.7%)	5730	(3.4%)	2547	(2.9%)	0.0565	0.0108
Black	107 478	76 100	(7.1%)	17 921	(6.2%)	9459	(5.6%)	3998	(4.6%)	0.1089	0.0213
Other	54 299	36 536	(3.4%)	8814	(3.1%)	5623	(3.3%)	3326	(3.8%)	0.0412	0.0036
Location											
Metro	1 268 421	802 314	(75.3%)	243 583	(84.7%)	146 638	(86.2%)	75 886	(87.1%)	0.3046	0.0814
Rural	40 672	33 224	(3.1%)	4231	(1.5%)	2257	(1.3%)	960	(1.1%)	0.1407	0.0546
urban	301 544	230 234	(21.6%)	39 832	(13.8%)	21 152	(12.4%)	10 326	(11.8%)	0.2637	0.0644
Education Percentile											
<86.6	403 498	285 232	(26.8%)	65 031	(22.6%)	35 802	(21.1%)	17 433	(20.0%)	0.1603	0.0386
86.6—91.4	404 501	274 391	(25.7%)	69 505	(24.2%)	40 504	(23.8%)	20 101	(23.1%)	0.0626	0.0055
91.5–94.8	401 823	261 470	(24.5%)	73 403	(25.5%)	44 031	(25.9%)	22 919	(26.3%)	0.0404	0.0125
>94.8		244 679	(23.0%)	79 707	(27.7%)	49 710	(29.2%)	26 719	(30.7%)	0.1743	0.0308
Original Entitlement											
OASI	400 815	951 682	(89.3%)	262 714	(91.3%)	157 223	(92.5%)	81 205	(93.2%)	0.1367	0.0317
DIB	1 452 824	111 688	(10.5%)	24 461	(8.5%)	12 596	(7.4%)	5887	(6.8%)	0.1331	0.0307
ESRD	1598	1203	(0.1%)	223	(0.1%)	129	(0.1%)	43	(0.0%)	0.0223	0.0055
DIB&ESRD	1583	1199	(0.1%)	248	(0.1%)	99	(0.1%)	37	(0.0%)	0.0252	0.0068
Dual Eligibility											
No	1 335 509	860 146	(80.7%)	247 122	(85.9%)	150 274	(88.4%)	77 967	(89.4%)	0.247	0.095
Yes	275 128	205 626	(19.3%)	40 524	(14.1%)	19 773	(11.6%)	9205	(10.6%)	0.247	0.095
Physical Inactivity											
<20.5	385 224	245 757	(23.1%)	72 844	(25.3%)	43 476	(25.6%)	23 147	(26.6%)	0.081	0.0208
20.5–24.2	396 187	250 569	(23.5%)	74 631	(25.9%)	46 367	(27.3%)	24 620	(28.2%)	0.1082	0.0191
24.3–27.6	414 175	272 308	(25.6%)	75 077	(26.1%)	44 465	(26.1%)	22 325	(25.6%)	0.0137	0.0075
>27.6	415 051	297 138	(27.9%)	65 094	(22.6%)	35 739	(21.0%)	17 080	(19.6%)	0.1957	0.0469
Associations Rate											
<7.2	369 785	238 274	(22.4%)	69 207	(24.1%)	40 716	(23.9%)	21 588	(24.8%)	0.0568	0.0082
7.2– 9.3	435 178	274 299	(25.7%)	84 351	(29.3%)	50 848	(29.9%)	25 680	(29.5%)	0.0931	0.0231
9.4–11.6	387 295	252 950	(23.7%)	70 369	(24.5%)	42 319	(24.9%)	21 657	(24.8%)	0.0269	0.0067
>11.6	418 379	300 249	(28.2%)	63 719	(22.2%)	36 164	(21.3%)	18 247	(20.9%)	0.1688	0.0384
Having PCP											
No	645 209	500 159	(46.9%)	89 150	(31.0%)	39 903	(23.5%)	15 997	(18.4%)	0.6399	0.1593
Yes	965 428	565 613	(53.1%)	198 496	(69.0%)	130 144	(76.5%)	71 175	(81.6%)	0.6399	0.1593
Neurologists Visit											
No	1 277 866	865 973	(81.3%)	220 480	(76.6%)	127 120	(74.8%)	64 293	(73.8%)	0.1803	0.0434
Yes	332 771	199 799	(18.7%)	67 166	(23.4%)	42 927	(25.2%)	22 879	(26.2%)	0.1803	0.0434
Psychiatrics Visit											
No	1 553 167	1 030 967	(96.7%)	275 856	(95.9%)	162 911	(95.8%)	83 433	(95.7%)	0.0537	0.0156
Yes	57 470	34 805	(3.3%)	11 790	(4.1%)	7136	(4.2%)	3739	(4.3%)	0.0537	0.0156
Number of Hospitalizations											
0	944 345	611 047	(57.3%)	170 184	(59.2%)	104 611	(61.5%)	58 503	(67.1%)	0.2027	0.0401
1	385 875	260 121	(24.4%)	68 251	(23.7%)	39 114	(23.0%)	18 389	(21.1%)	0.0791	0.0174
2	155 873	106 960	(10.0%)	27 587	(9.6%)	15 069	(8.9%)	6257	(7.2%)	0.102	0.017
3	66 443	46 171	(4.3%)	11 619	(4.0%)	6293	(3.7%)	2360	(2.7%)	0.0883	0.0171
4+	58 101	41 473	(3.9%)	10 005	(3.5%)	4960	(2.9%)	1663	(1.9%)	0.1184	0.0262
Tobacco											
No	1 482 494	976 183	(91.6%)	265 796	(92.4%)	158 485	(93.2%)	82 030	(94.1%)	0.0974	0.0192
Yes	128 143	89 589	(8.4%)	21 850	(7.6%)	11 562	(6.8%)	5142	(5.9%)	0.0974	0.0192
Osteoporosis											
No	1 301 923	875 387	(82.1%)	227 877	(79.2%)	131 642	(77.4%)	67 017	(76.9%)	0.1305	0.0219
Yes	308 714	190 385	(17.9%)	59 769	(20.8%)	38 405	(22.6%)	20 155	(23.1%)	0.1305	0.0219
Previous Fracture											
No	1 371 937	904 811	(84.9%)	244 717	(85.1%)	145 953	(85.8%)	76 456	(87.7%)	0.0818	0.0136
Yes	238 700	160 961	(15.1%)	42 929	(14.9%)	24 094	(14.2%)	10 716	(12.3%)	0.0818	0.0136
Previous Fall											
No	1 266 898	834 676	(78.3%)	225 355	(78.3%)	135 214	(79.5%)	71 653	(82.2%)	0.0976	0.0199
Yes	343 739	231 096	(21.7%)	62 291	(21.7%)	34 833	(20.5%)	15 519	(17.8%)	0.0976	0.0199

We further included follow-up AWV as a time-dependent variable. The AWV status can be transferred to a non-AWV status, and the non-AWV status can be transferred to an AWV status in the follow-up period through the end of study (12/31/2021).

We examined whether AWVs modified the disparity in fall or fracture across severity of ADRD, sex, race/ethnicity, education and rural/urban residence by adding the interaction between the number of AWVs and each variable in a Cox proportional hazards model adjusted for all covariables in Table 1. We further assessed whether AWV’s effect on outcomes varied by previous falls, previous fractures and osteoporosis by adding the interactions in the model. All analyses were performed using SAS Enterprise version 7.1 (SAS Institute, Cary, NC).

## Results

### AWV utilisation and patient characteristics

As shown in Table 1, among the 1 610 637 Medicare beneficiaries aged ≥68 years with ADRD, 66.2% received no AWVs in the three years prior to the index date (2017), while 17.9% received one, 10.6% received two and 5.4% received three or more AWVs. Patient characteristics varied across AWV utilisation levels. Those with ≥3 AWVs were more likely to have a PCP (81.6% vs. 53.1%), seen a neurologist (26.2% vs. 18.7%) and have mild rather than moderate/severe ADRD (76.3% vs. 63.9%). Additionally, they were less likely to have Medicare dual enrolment status (10.6% vs. 19.3%), and more likely to have had no hospitalizations in the prior year (67.1% vs. 57.3%).

### Fall outcomes

**Figure 1:**
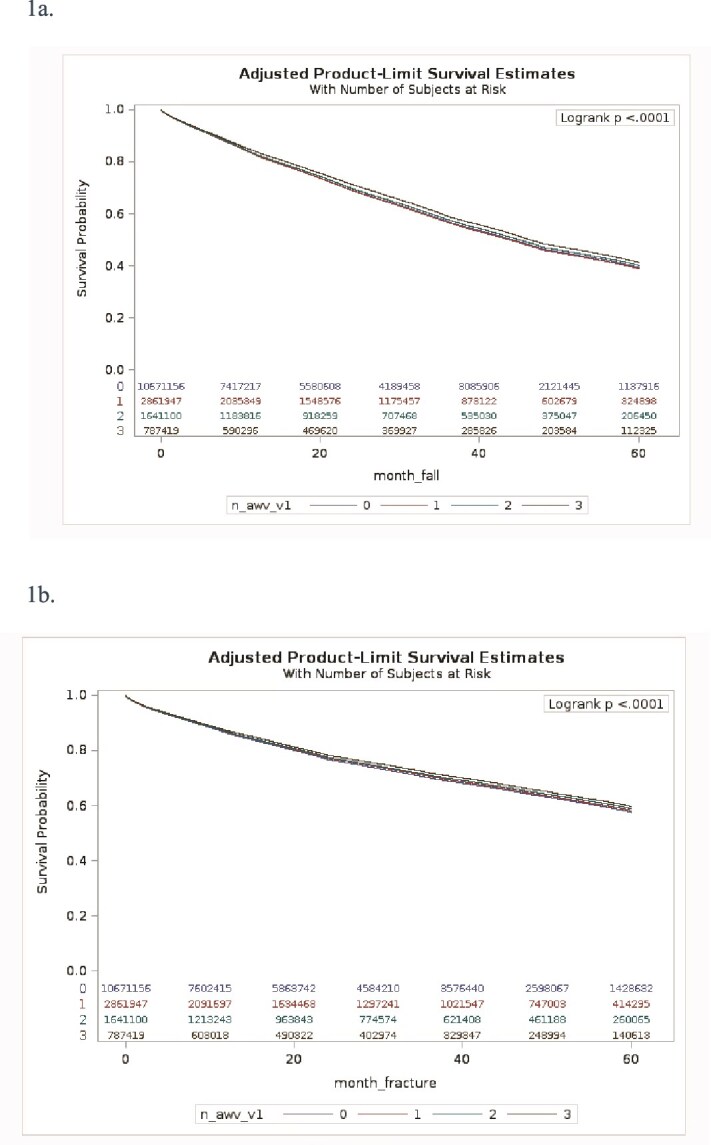
(a) Kaplan–Meier Fall-Free Survival by AWV Frequency. (b) Kaplan–Meier Fracture-Free Survival by AWV Frequency.

In Figure 1a, Kaplan–Meier estimates demonstrated a dose–response relationship between AWV participation and fall risk. At 2 years of follow-up, the cumulative incidence of falls was 30.98%, 31.08%, 30.33% and 28.74% for patients with 0, 1, 2 and ≥ 3 AWVs, respectively. At 5 years, the cumulative incidence of falls was 60.59%, 61.04%, 59.85% and 58.78% across the same groups. Table 2 shows Cox proportional hazards models with IPTW, the HRs for falls were 1.008 (95% CI: 1.006–1.010) for one AWV, 0.976 (95% CI: 0.973–0.978) for two AWVs and 0.936 (95% CI: 0.933–0.939) for ≥3 AWVs, compared to beneficiaries with no AWVs. Examining time dependency effects found in Table 2, follow-up AWVs after the index year were associated with an additional 7.9% reduction in fall risk (HR: 0.921; 95% CI: 0.920–0.923), indicating continued AWVs is associated with more protective benefits.

### Fracture outcomes

A similar but less defined trend was seen for fractures demonstrated by Kaplan–Meier estimates. As shown in Figure 1b, Kaplan–Meier estimates for fractures showed a less pronounced but consistent decline in cumulative incidence with increasing AWV exposure. At 2 years, the cumulative incidence of fractures was 23.30%, 22.78%, 22.53% and 21.74% for patients with 0, 1, 2 and ≥ 3 AWVs, respectively. At 5 years, the rates were 42.45%, 41.85%, 41.16% and 40.39% across the same groups. Compared to non-recipients, the adjusted HRs for fractures in Table 2 were 0.978 (95% CI: 0.975–0.980) for one AWV, 0.960 (95% CI: 0.957–0.963) for two AWVs and 0.927 (95% CI: 0.923–0.931) for ≥3 AWVs. Table 2 also reveals that a follow-up AWV in the post-index period was associated with a 7.1% lower risk of fracture (HR: 0.929; 95% CI: 0.926–0.931). Like falls, there is a dose dependent response noted for fracture.

### Interaction effects and subgroup analysis

#### Subgroup analysis for falls

Subgroup interaction analyses for fall outcomes in Table 3 showcased that AWV effects varied across many characteristics including: ADRD severity, sex, race and education level. Among patients with mild ADRD, AWVs were associated with a notable reduction in fall risk, with a HR of 0.911 (95% CI: 0.900–0.922) for those receiving ≥3 AWVs, compared to a HR of 0.988 (95% CI: 0.968–1.009) in patients with severe ADRD. Male beneficiaries showed a stronger effect from ≥3 AWVs than females, with HRs of 0.908 (95% CI: 0.892–0.924) and 0.937 (95% CI: 0.924–0.950), respectively. Racial subgroup analyses revealed a significant reduction in fall risk among non-Hispanic White (HR: 0.922; 95% CI: 0.912–0.933) and Other race groups (HR: 0.916; 95% CI: 0.860–0.976), which was not as prominent in Black and Hispanic populations. Educational level also impacted AWV effectiveness, with patients in the highest education group (>94.9 percentile) experiencing the greatest benefit (HR: 0.912; 95% CI: 0.894–0.929), compared to those in the lowest group (<86.8 percentile, HR: 0.945; 95% CI: 0.922–0.968). Comparing with patients who did not have previous falls, AWVs were associated with higher reduction of fall among those who had previous falls.

**Table 2:** Association between AWV frequency and risk of falls and fractures using post-IPTW and time-dependent cox models.

Model	AWV	Fall		Fracture	
		HR	95% CI	HR	95% CI
Post IPTW Cox Model					
	0	REF		REF	
	1	1.008	(1.006–1.010)	0.978	(0.975–0.980)
	2	0.976	(0.973–0.978)	0.960	(0.957–0.963)
	≥3	0.936	(0.933–0.939)	0.927	(0.923–0.931)
Time Dependent Model Previous AWV					
	0	REF		REF	
	1	1.031	(1.029–1.033)	0.999	(0.996–1.001)
	2	1.013	(1.010–1.015)	0.994	(0.990–0.997)
	≥3	0.985	(0.982–0.989)	0.973	(0.969–0.978)
Future AWV					
	0	REF		REF	
	1	0.921	(0.920–0.923)	0.929	(0.926–0.931)
					

**Table 3:** Interaction effects on fall risk by AWVs frequency.

Characteristic	AWV 1 vs. 0		AWV 2 vs. 0		AWV 3+ vs. 0	
	HR	95% CI	HR	95% CI	HR	95% CI
ADRD Severity						
Severe	1.027	(1.016–1.039)	0.999	(0.985–1.014)	0.988	(0.968–1.009)
Mild	0.999	(0.991–1.007)	0.961	(0.951–0.970)	0.911	(0.900–0.922)
Sex						
Female	1.009	(1.002–1.017)	0.975	(0.966–0.985)	0.937	(0.924–0.950)
Male	1.003	(0.992–1.014)	0.958	(0.945–0.971)	0.908	(0.892–0.924)
Race						
White	1.010	(1.003–1.017)	0.970	(0.962–0.978)	0.922	(0.912–0.933)
Hispanic	1.012	(0.976–1.050)	1.019	(0.973–1.067)	1.008	(0.943–1.077)
Black	0.984	(0.955–1.013)	0.964	(0.928–1.002)	0.994	(0.941–1.051)
Other	0.977	(0.938–1.017)	0.944	(0.899–0.992)	0.916	(0.860–0.976)
Education Percentile						
<86.8	1.001	(0.988–1.015)	0.969	(0.951–0.986)	0.945	(0.922–0.968)
86.8–91.5	1.005	(0.992–1.018)	0.972	(0.956–0.988)	0.935	(0.915–0.956)
91.6–94.9	1.010	(0.997–1.022)	0.970	(0.955–0.985)	0.919	(0.900–0.938)
>94.9	1.011	(0.999–1.023)	0.966	(0.952–0.980)	0.912	(0.894–0.929)
Previous fall						
*No*	1.007	(1.000–1.015)	0.968	(0.959–0.977)	0.920	(0.909–0.931)
*Yes*	1.010	(0.997–1.022)	0.979	(0.964–0.995)	0.957	(0.936–0.978)

#### Subgroup analyses for fractures

Subgroup analyses for fractures were also conducted, as shown in Table 4. Among patients with mild ADRD, the HR at ≥3 AWVs was 0.902 (95% CI: 0.888–0.916), while those with severe ADRD had a HR of 0.980 (95% CI: 0.955–1.004). Male beneficiaries, with an HR of 0.894 (95% CI: 0.873–0.915), exhibited stronger benefit compared to females, with an HR of 0.928 (95% CI: 0.913–0.942), at ≥3 AWVs. Non-Hispanic White beneficiaries had an HR of 0.916 (95% CI: 0.904–0.929), and Other race individuals had a similar reduction with an HR of 0.876 (95% CI: 0.811–0.946). In contrast, Black beneficiaries showed no significant benefit from AWV participation, with an HR of 1.000 (95% CI: 0.924–1.082) at ≥3 AWVs. Additionally, Hispanic beneficiaries had received protective effects at ≥3 AWVs with a HR of 0.918 (95% CI: 0.845–0.996). Similar to the findings on fall, patients without previous fracture or without osteoporosis had larger reduction of fracture associated with AWVs.

**Table 4:** Interaction effects on fracture risk by AWV frequency.

**Characteristic**	AWV 1 vs. 0		AWV 2 vs. 0		AWV 3+ vs. 0	
	**HR**	95% CI	HR	95% CI	HR	95% CI
ADRD Severity						
Severe	0.996	(0.983–1.009)	0.981	(0.965–0.998)	0.980	(0.955–1.004)
Mild	0.969	(0.960–0.978)	0.947	(0.936–0.958)	0.902	(0.888–0.916)
Sex						
Female	0.980	(0.971–0.990)	0.959	(0.948–0.970)	0.928	(0.913–0.942)
Male	0.967	(0.953–0.982)	0.942	(0.925–0.959)	0.894	(0.873–0.915)
Race						
White	0.978	(0.970–0.986)	0.958	(0.948–0.968)	0.916	(0.904–0.929)
Hispanic	0.973	(0.932–1.017)	0.928	(0.877–0.982)	0.918	(0.845–0.996)
Black	0.998	(0.956–1.041)	0.940	(0.889–0.994)	1.000	(0.924–1.082)
Other	0.920	(0.876–0.966)	0.910	(0.858–0.966)	0.876	(0.811–0.946)
Location						
Metro	0.978	(0.970–0.987)	0.959	(0.949–0.969)	0.918	(0.905–0.931)
Rural	1.038	(0.977–1.102)	0.840	(0.813–0.961)	0.936	(0.830–1.056)
Urban	0.965	(0.946–0.985)	0.937	(0.912–0.963)	0.916	(0.883–0.951)
Education Percentile						
<86.8	0.960	(0.944–0.976)	0.953	(0.932–0.974)	0.919	(0.892–0.947)
86.8–91.5	0.972	(0.957–0.988)	0.954	(0.936–0.974)	0.928	(0.903–0.954)
91.6–94.9	0.978	(0.963–0.993)	0.954	(0.936–0.972)	0.896	(0.874–0.919)
>94.9	0.992	(0.978–1.007)	0.957	(0.940–0.974)	0.925	(0.904–0.947)
Osteoporosis						
*No*	0.975	(0.966–0.984)	0.946	(0.935–0.957)	0.903	(0.889–0.918)
*Yes*	0.980	(0.965–0.994)	0.971	(0.954–0.989)	0.943	(0.921–0.966)
Previous fracture						
*No*	0.980	(0.971–0.989)	0.952	(0.941–0.962)	0.910	(0.897–0.924)
*Yes*	0.969	(0.955–0.984)	0.966	(0.947–0.984)	0.950	(0.924–0.975)

## Discussion

Among the 1,610,637 Medicare beneficiaries aged ≥68 years with ADRD, more AWV participation was associated with lower risks of falls and fractures. A dose–response relationship of AWV effect on falls and fractures was observed, with the most benefit seen in participants with three or more AWVs. Follow-up serial AWVs led to reduction in risk for both falls and fractures. Subgroup analyses indicated that AWV benefits were particularly impactful among ADRD patients with the following characteristics: mild ADRD, males, non-Hispanic White or Other race, and those with higher education levels. Notably, Hispanic patients did experience a significant reduction in fracture risk with increased AWV exposure, but these results did not extend to reduction falls. The reason for this is unclear; future studies are needed to examine subgroup differences among ADRD patients who got AWV in the rates of receipt of osteoporosis medications and referral to physical therapy. Among Black patients, AWVs were not associated with a statistically significant reduction in fractures. Reasons for Black-White differences in impact of AWV on fracture are unclear but potential explanations include differences in rate of osteoporosis and its treatments and social determinants of health among black populations [14]. Further studies are required to further understand racial differences and AWV effectiveness.

Our results are consistent with research findings from prior regional study showing association of AWVs with lower odds of fall and fracture in general population of older adults. Our national study of Medicare beneficiaries confirmed these findings and expanded them to older adults living with ADRD.

From a clinical perspective, our results underscore the need for interventions to increase the long-term and consistent AWV use by clinicians, especially among subgroups (e.g. rural residents and racial minorities) where less than expected benefits were observed. Our findings also highlight the value of primary care relationships in facilitating AWV participation; beneficiaries with an established PCP were significantly more likely to receive AWVs. Strengthening the PCP-patient relationship may be a potential strategy to increase AWV utilisation and its benefits. Our findings align with current studies, which have shown that patients with PCPs are more likely to receive preventative care interventions [15].

Previous studies have also reported that AWV utilisation and its benefits may differ across geographical populations [16, 17]. We found that AWV participation was associated with fall and fracture risk reductions for metro and urban residents. However, AWV risk reduction effects in rural residents were weaker for falls, and results were not statistically significant for fractures. This is an area for future study to understand and identify policy/practise-actionable factors associated with rural–urban disparity in AWV use and outcomes.

## Limitations

This study has a limitation because the cohort includes only fee for service Medicare beneficiaries. Thus, our findings may not be applicable to those in Medicare advantage or Medicare HMOs. Another limitation is potential for both ADRD misdiagnosis [18, 19] and more commonly missed diagnosis of early ADRD. Claims-based diagnosis of ADRD using CMS CCW might not fully and accurately capture the full range of ADRD (MCI, mild dementia to severe dementia) as early dementia stages are under documented in claims database due to under-recognition and under-diagnosis of cognitive impairment in general Medicare population [20]. This limitation is due to under-recognition in clinical practise since our outcomes were derived from medically documented billing encounters rather than patient self-reports. Therefore, ADRD misclassification, due to the nature of claims data, is expected to weaken, rather than strengthen, observed associations between AWVs and fall or fracture risk in our study. We partially compensated for this by using frailty index to capture severity of dementia. Additionally, in our study we did not include Medicare Part D data. Thus, we cannot examine the effects of Fall Risk Increasing Drugs (FRIDs), other osteoporosis medications and medication changes with AWVs. Future studies should use Medicare Part D data to explore if FRID, medication management and osteoporosis medicine are potential mechanisms behind AWV effectiveness [21, 22]. Another limitation of claims data is that does not provide details, such as the cause of each fracture; therefore, we cannot distinguish fractures caused by falls from other fractures. However, our study is strictly focusing on if the AWV itself reduces falls and fractures for older adults with ADRD. We encourage future studies to explore mechanisms behind AWV by using more granular data. Another limitation of claims data is that minor falls are under reported relative to fractures. Another potential source of underreporting of falls in the ADRD population relates to the nature of cognitive impairment and memory loss associated with ADRD; this would introduce recall bias as patients may simply not remember episodes of falls, which thus lead to under-reporting, unless the falls are witnessed and reported by caregivers. We also did not capture how older patients with ADRD subsequently (after AWV) use guideline recommended interventions [23] aimed at directly preventing falls (e.g. PT/OT use, home modifications, elimination of drugs with high fall risk, etc.) and fractures (use of osteoporosis medications)—clearly an area for future study.

## Future research

Future research should investigate barriers and facilitators to AWV effectiveness in vulnerable populations, especially rural residents. Specifically, we should explore the mechanisms behind subgroup differences and evaluate targeted approaches, such as digital interventions and telehealth, to improve AWV uptake and its effectiveness across all demographic groups [24, 25]. Although there are current studies that have explored ways to increase AWV participation, additional research should examine how to best improve AWV for various subgroups based on demographics and social determinants of health in the ADRD population [26, 27]. Additionally, due to rise of telehealth, there should be further studies comparing effectiveness of virtual versus in-person AWVs, especially for assessing AWV impact on fall and fracture risk [25]. While current studies have investigated the usage of cognitive assessments during AWVs, there can be more research investigating rates, patterns and outcome of referral to rehabilitation specialists (e.g. PT) and prescribing of osteoporosis medications after fall and fracture risk assessment during AWV [28]. These efforts may lead to more specific interventions and policies to enhance AWV impact on patient-relevant outcomes (e.g. falls, fractures, premature disability and institutionalisation) in the growing populations of older patients with ADRD.

## Supplementary Material

afag065_aa-25-2339-File002
