# Supplementary material for: Impact of annual wellness visits on preventing falls and fractures for Alzheimer’s disease and related dementias older adults
Source: Age Ageing. 2026 Mar 30;55(3):afag065. doi: 10.1093/ageing/afag065 (PMC13034919; doi:10.1093/ageing/afag065)
Supplement: afag065_aa-25-2339-File002 [file afag065_aa-25-2339-file002.docx]

**Impact of Annual Wellness Visits on Preventing Falls and Fractures for Alzheimer’s Disease and Related Dementias Older Adults**

**SUPPLEMENTAL TABLE 1**

Codes for a fracture or fall from the *International Statistical Classification of Diseases and Related Health Problems,* 10th Revision (ICD-10)

| **Variable** | **Details** | |
| --- | --- | --- |
| *Hip/pelvic fracture* | | |
|  | *Source:* MedPAR, Outpatient Standard Analytical files, Carrier file | |
|  | *Definition:* ICD-10/CPT4/HCPCS diagnosis codes | |
|  |  | M80.051A, M80.052A, M80.059A, M80.851A, M80.852A, M80.859A, M84.350A, M84.351A, M84.352A, M84.353A, M84.359A, M84.451A, M84.452A, M84.453A, M84.459A, M84.550A, M84.551A, M84.552A, M84.553A, M84.559A, M84.650A, M84.651A, M84.652A, M84.653A, M84.659A, S32.301A, S32.301B, S32.302A, S32.302B, S32.309A, S32.309B, S32.311A, S32.311B, S32.312A, S32.312B, S32.313A, S32.313B, S32.314A, S32.314B, S32.315A, S32.315B, S32.316A, S32.316B, S32.391A, S32.391B, S32.392A, S32.392B, S32.399A, S32.399B, S32.401A, S32.401B, S32.402A, S32.402B, S32.409A, S32.409B, S32.411A, S32.411B, S32.412A, S32.412B, S32.413A, S32.413B, S32.414A, S32.414B, S32.415A, S32.415B, S32.416A, S32.416B, S32.421A, S32.421B, S32.422A, S32.422B, S32.423A, S32.423B, S32.424A, S32.424B, S32.425A, S32.425B, S32.426A, S32.426B, S32.431A, S32.431B, S32.432A, S32.432B, S32.433A, S32.433B, S32.434A, S32.434B, S32.435A, S32.435B, S32.436A, S32.436B, S32.441A, S32.441B, S32.442A, S32.442B, S32.443A, S32.443B, S32.444A, S32.444B, S32.445A, S32.445B, S32.446A, S32.446B, S32.451A, S32.451B, S32.452A, S32.452B, S32.453A, S32.453B, S32.454A, S32.454B, S32.455A, S32.455B, S32.456A, S32.456B, S32.461A, S32.461B, S32.462A, S32.462B, S32.463A, S32.463B, S32.464A, S32.464B, S32.465A, S32.465B, S32.466A, S32.466B, S32.471A, S32.471B, S32.472A, S32.472B, S32.473A, S32.473B, S32.474A, S32.474B, S32.475A, S32.475B, S32.476A, S32.476B, S32.481A, S32.481B, S32.482A, S32.482B, S32.483A, S32.483B, S32.484A, S32.484B, S32.485A, S32.485B, S32.486A, S32.486B, S32.491A, S32.491B, S32.492A, S32.492B, S32.499A, S32.499B, S32.501A, S32.501B, S32.502A, S32.502B, S32.509A, S32.509B, S32.511A, S32.511B, S32.512A, S32.512B, S32.519A, S32.519B, S32.591A, S32.591B, S32.592A, S32.592B, S32.599A, S32.599B, S32.601A, S32.601B, S32.602A, S32.602B, S32.609A, S32.609B, S32.611A, S32.611B, S32.612A, S32.612B, S32.613A, S32.613B, S32.614A, S32.614B, S32.615A, S32.615B, S32.616A, S32.616B, S32.691A, S32.691B, S32.692A, S32.692B, S32.699A, S32.699B, S32.810A, S32.810B, S32.811A, S32.811B, S32.82XA, S32.82XB, S32.89XA, S32.89XB, S32.9XXA, S32.9XXB, S72.001A, S72.001B, S72.001C, S72.002A, S72.002B, S72.002C, S72.009A, S72.009B, S72.009C, S72.011A, S72.011B, S72.011C, S72.012A, S72.012B, S72.012C, S72.019A, S72.019B, S72.019C, S72.021A, S72.021B, S72.021C, S72.022A, S72.022B, S72.022C, S72.023A, S72.023B, S72.023C, S72.024A, S72.024B, S72.024C, S72.025A, S72.025B, S72.025C, S72.026A, S72.026B, S72.026C, S72.031A, S72.031B, S72.031C, S72.032A, S72.032B, S72.032C, S72.033A, S72.033B, S72.033C, S72.034A, S72.034B, S72.034C, S72.035A, S72.035B, S72.035C, S72.036A, S72.036B, S72.036C, S72.041A, S72.041B, S72.041C, S72.042A, S72.042B, S72.042C, S72.043A, S72.043B, S72.043C, S72.044A, S72.044B, S72.044C, S72.045A, S72.045B, S72.045C, S72.046A, S72.046B, S72.046C, S72.051A, S72.051B, S72.051C, S72.052A, S72.052B, S72.052C, S72.059A, S72.059B, S72.059C, S72.061A, S72.061B, S72.061C, S72.062A, S72.062B, S72.062C, S72.063A, S72.063B, S72.063C, S72.064A, S72.064B, S72.064C, S72.065A, S72.065B, S72.065C, S72.066A, S72.066B, S72.066C, S72.091A, S72.091B, S72.091C, S72.092A, S72.092B, S72.092C, S72.099A, S72.099B, S72.099C, S72.101A, S72.101B, S72.101C, S72.102A, S72.102B, S72.102C, S72.109A, S72.109B, S72.109C, S72.111A, S72.111B, S72.111C, S72.112A, S72.112B, S72.112C, S72.113A, S72.113B, S72.113C, S72.114A, S72.114B, S72.114C, S72.115A, S72.115B, S72.115C, S72.116A, S72.116B, S72.116C, S72.121A, S72.121B, S72.121C, S72.122A, S72.122B, S72.122C, S72.123A, S72.123B, S72.123C, S72.124A, S72.124B, S72.124C, S72.125A, S72.125B, S72.125C, S72.126A, S72.126B, S72.126C, S72.131A, S72.131B, S72.131C, S72.132A, S72.132B, S72.132C, S72.133A, S72.133B, S72.133C, S72.134A, S72.134B, S72.134C, S72.135A, S72.135B, S72.135C, S72.136A, S72.136B, S72.136C, S72.141A, S72.141B, S72.141C, S72.142A, S72.142B, S72.142C, S72.143A, S72.143B, S72.143C, S72.144A, S72.144B, S72.144C, S72.145A, S72.145B, S72.145C, S72.146A, S72.146B, S72.146C, S72.21XA, S72.21XB, S72.21XC, S72.22XA, S72.22XB, S72.22XC, S72.23XA, S72.23XB, S72.23XC, S72.24XA, S72.24XB, S72.24XC, S72.25XA, S72.25XB, S72.25XC, S72.26XA, S72.26XB, S72.26XC, S79.001A, S79.002A, S79.009A, S79.011A, S79.012A, S79.019A, S79.091A, S79.092A, S79.099A as the primary diagnosis from inpatient or outpatient claims^a^ |
| *Other fractures (including orbital fractures)* | | |
|  | *Source:* MedPAR, Outpatient Standard Analytical files, Carrier file | |
|  | *Definition:* ICD-10 diagnosis codes | |
|  |  | S02.3X, S02.83X, S02.84X, S12X, S22X, S32X, S42X, S52X, S62X, S72.3–S72.9X, S82X, S92X as the primary diagnosis from inpatient or outpatient claims^b^ |
| *Fall mechanism (any diagnosis positions)* | | |
|  | *Source:* MedPAR, Outpatient Standard Analytical files, Carrier file | |
|  | *Definition:* ICD-10 diagnosis codes (excluding W03X, W04X ICD 10 codes for fall involving another person in Chen A, Canner JK, Zafar S, et al. paper ^b^ Supp. Table 1) | |
|  |  | W00X, W01X, W05X–W19X, V0011X, V0013X, V0014X, V0015X, V0031X, V0032X at any diagnosis position from inpatient or outpatient or carrier claims^b,c^ |

^a^CMS chronic condition warehouse (CCW) CCW condition algorithms: hip/pelvic fracture. Centers for Medicare and Medicaid Services. Last reviewed February 2021. Accessed December 6, 2021. https://www2.ccwdata.org/documents/10280/19139608/ccw-cond-algo-hipfracture.pdf

^b^Chen A, Canner JK, Zafar S, et al. Characteristics of ophthalmic trauma in fall-related hospitalizations in the united states from 2000 to 2017. *Ophthalmic Epidemiol*. 2021;29(2):206-215. doi:10.1080/09286586.2021.1914668

^c^Shah R, Raji MA, Westra J, Kuo YF. Association of co-prescribing of opioid and benzodiazepine substitutes with incident falls and fractures among older adults: a cohort study. *BMJ Open*. 2021;11(12):e052057. doi:10.1136/bmjopen-2021-052057

MedPAR = Medicare Provider Analysis and Review; CPT4 = *Current Procedural Terminology,* Fourth Edition; HCPCS = Healthcare Common Procedure Coding System.
